# *In Vivo* Immunotoxicity of SiO2@(Y0.5Gd0.45Eu0.05)2O3 as Dual-Modality Nanoprobes

**DOI:** 10.3390/ijms150813649

**Published:** 2014-08-07

**Authors:** Xiumei Tian, Ermao Li, Fanwen Yang, Ye Peng, Jixiang Zhu, Fupo He, Xiaoming Chen

**Affiliations:** Department of Biomedical Engineering, Guangzhou Medical University, Guangzhou 510182, China; E-Mails: tianxiumei224@gmail.com (X.T.); bailang74624@gmail.com (E.L.); yangfanwen42@gmail.com (F.Y.); pengyejlu00@gmail.com (Y.P.); zhujixiang888@gmail.com (J.Z.); wild.4145@gmail.com (F.H.)

**Keywords:** immunotoxicity, reactive oxygen species, nanoprobe, gadolinium, europium, molecular imaging

## Abstract

We have successfully synthesized SiO_2_@(Y_0.5_Gd_0.45_Eu_0.05_)_2_O_3_ nanocomposites as a potential dual-modality nanoprobe for molecular imaging *in vitro*. However, their immunotoxicity assessment *in vivo* remains unknown. In this article, the *in vitro* biocompatibility of our dual-modality nanoprobes was assayed in terms of cell viability and apoptosis. *In vivo* immunotoxicity was investigated by monitoring the generation of reactive oxygen species (ROS), cluster of differentiation (CD) markers and cytokines in Balb/c mice. The data show that the *in vitro* biocompatibility was satisfactory. In addition, the immunotoxicity data revealed there are no significant changes in the expression levels of CD11b and CD71 between the nanoprobe group and the Gd in a diethylenetriaminepentaacetic acid (DTPA) chelator (Gd-DTPA) group 24 h after injection in Balb/c mice (*p* > 0.05). Importantly, there are significant differences in the expression levels of CD206 and CD25 as well as the secretion of IL-4 and the generation of ROS 24 h after injection (*p* < 0.05). Transmission electron microscopy (TEM) images showed that few nanoprobes were localized in the phagosomes of liver and lung. In conclusion, the toxic effects of our nanoprobes may mainly result from the aggregation of particles in phagosomes. This accumulation may damage the microstructure of the cells and generate oxidative stress reactions that further stimulate the immune response. Therefore, it is important to evaluate the *in vivo* immunotoxicity of these rare earth-based biomaterials at the molecular level before molecular imaging *in vivo*.

## 1. Introduction

Rare earth-based nano-materials have attracted considerable attention due to their specific physicochemical characteristics in molecular imaging and medical diagnosis [1,2,3]. They also may do potential harm to human health because of their high surface-to-volume ratios, small size, different shapes, positive surface charges, *etc.* Thus, risk assessment of these materials is a particularly important scientific issue [4,5]. While toxicity assessments of rare earth-based nanomaterials are common *in vitro*, *in vivo* toxicity data remains scarce [4,6,7].

As a highly reactive chemical, reactive oxygen species (ROS) are widely related to physiological and pathological phenomena. Overproduction of ROS is an important mechanism in nanotoxicity [8,9]. ROS is a critical regulator of immunity, and the expression of cluster of differentiation (CD) markers correlates to activated or proliferated immune cells. Cytokines also play a crucial role in modulating the immune response. Besides cellular oxidative stress data, the generation of ROS *in vivo* has not been studied extensively especially for rare earth-based nanomaterials. ROS are important regulators and suppressors of immune response [10,11]. The immune system protects the host from foreign substances including nano-based biomaterials. It is crucial to measure ROS and immunotoxicity *in vivo* to understand the relationship between the properties of rare earth-based nanomaterials and toxicity. Unfortunately, there are few available studies on the immunotoxicity of nano-based biomaterials *in vivo* [6,12].

In our previous research [13], we have successfully synthesized SiO_2_@(Y_0.__5_Gd_0.45_Eu_0.05_)_2_O_3_ nanocomposites as a potential dual-modality nanoprobe for magnetic resonance (MR) and optical imaging *in vitro*, but have yet to perform systematic assessments of its immunotoxicity *in vivo*. In this study, we systematically evaluate the toxicity of our dual-modality nanoprobes including cell viability and apoptosis *in vitro* as well as the generation of ROS in peripheral blood neutrophils. We further characterize the cluster of differentiation (CD) markers in peripheral blood as well as representative cytokines in the serum of Balb/c mice. These *in vivo* studies are important to assess the risk assessment of these dual-modality nanoprobes before clinical use.

## 2. Results and Discussion

### 2.1. Characterization of the Dual-Modality Nanoprobes

**Figure 1:**
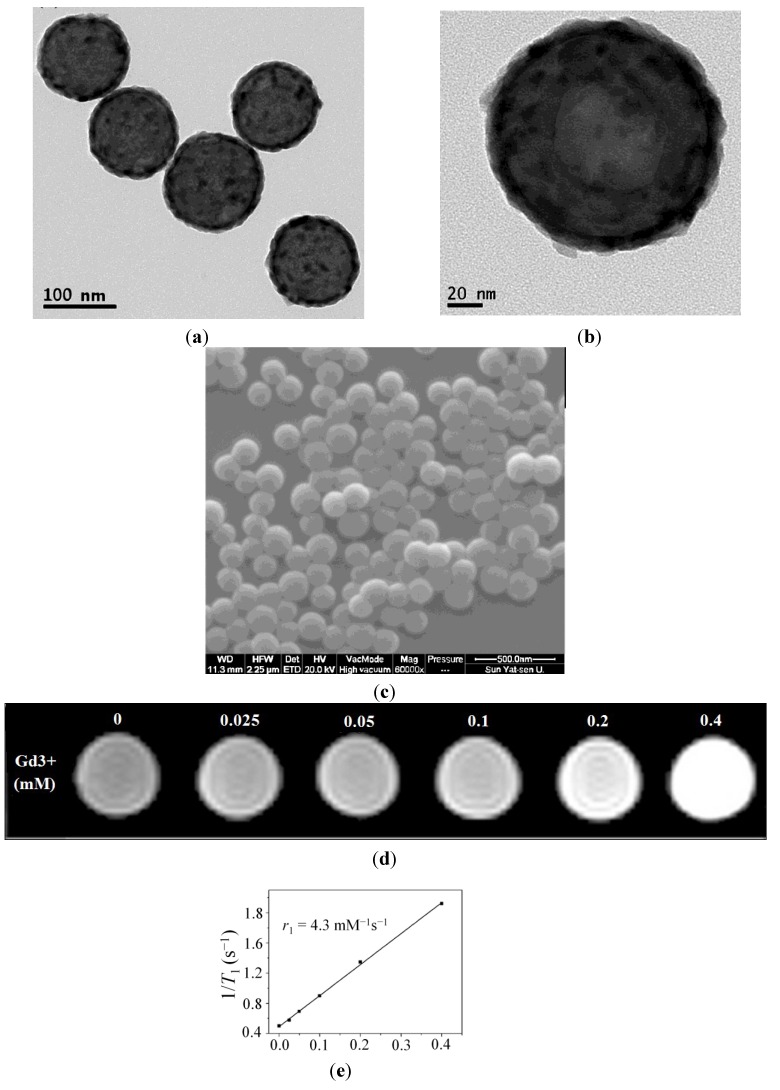
Characterization of the dual-modality nanoprobes. (**a**,**b**) transmission electron microscopy (TEM) micrograph and typical histograms for the dual-modality nanoprobes and SEM (**c**); (**d**) *T*_1_-weighted phantom MR image of the nanoprobes with Gd^3+^ concentrations of 0.025, 0.05, 0.1, 0.2 and 0.4 mM at 1.5 T; and (**e**) The relaxivity (*r*_1_) of the dual-modality nanoprobes by nuclear magnetic relaxation dispersion (NMRD) was at 1.5 T.

The microstructures and morphologies of the dual-modality nanoprobes were characterized by transmission electron microscopy (TEM) and scanning electron microscope (SEM). The typical TEM and SEM images (Figure 1a–c) show spherical nanoparticles with core–shell morphologies. The samples had good dispersion and uniformity. Furthermore, the *in vitro T*_1_-weighted MR images demonstrated that the signal intensity of the dual-modality nanoprobes enhanced with increasing Gd^3+^ concentration and the relaxivity (*r*_1_) is 4.3 mM^−1^·s^−1^ (Figure 1d,e). This suggests that our dual-modality nanoprobes are suitable for *T*_1_-weighted contrast agents *in vitro*. Additional characterization has been reported previously [13]. In addition, zeta potential of the dual-modality nanoprobes was (−7.31 ± 1.45) mV (Figure S1). This result suggested that it was difficult for the nanoprobes to combine with most proteins due to negative charge in the blood. To some extent, it is good for nanoprobes to decrease the potential toxicity.

### 2.2. Toxicity Effects on Macrophages in Vitro

**Figure 2:**
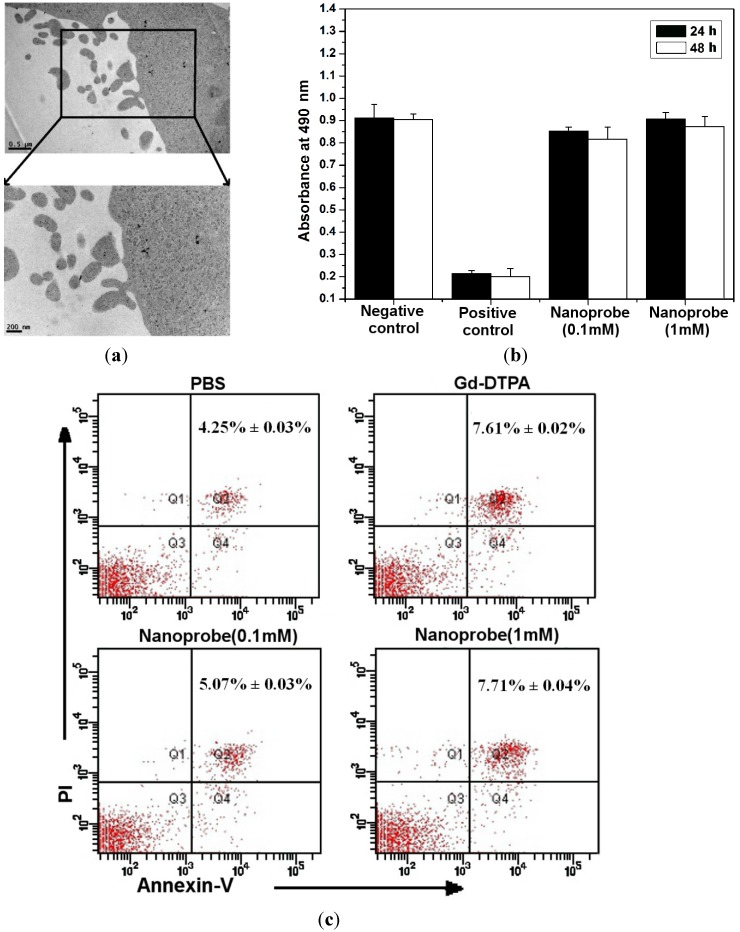
The *in vitro* biocompatibility of the dual-modality nanoprobes. (**a**) Cell uptake of dual-modality nanoprobes and TEM images of Raw 264.7 cells at 12 h; (**b**) Cell viability of the murine macrophage line Raw 264.7 cells incubated with different concentrations (0.1 and 1 mM) of the dual-modality nanoprobes at 24 and 48 h; and (**c**) Apoptosis rate of Raw264.7 cells were measured by flow cytometry 48 h after incubation of PBS, Gd-DTPA (1 mM) or the dual-modality nanoprobes (0.1 and 1 mM). Samples were stained by Annexin V and Propidium Iodide (PI).

In general, nano-based biomaterials are mainly removed by macrophages [14], and the macrophages are more sensitive to nanoparticle exposure than others cells [15]. TEM of the murine macrophage line Raw264.7 cells were used to evaluate cell uptake. As shown in Figure 2a, few nanoprobes were absorbed by Raw264.7 cells at 12 h after incubation with dual-modality nanoprobes (100 μM/L) and localized in phagosome. The size, zeta potential and coating of nanoprobe play significant role to affect on its cellular uptake. In addition, the viability of murine macrophage cell line Raw264.7 was evaluated by the MTT assay. Figure 2b shows that the cytotoxicity of Raw264.7 cells had no significant difference after incubation for 24 and 48 h (*p* > 0.05). This complements cytotoxicity data for L929 and lymphocyte cells from our precious study [13]. All cytotoxicity of the nanoprobes was negligible and indicated that the concentration of free Gd^3+^ ions was low.

Apoptosis relates to risk assessment *in vitro* [16]. To further analyze the cytotoxicity, we detected the apoptosis of Raw264.7 cells after incubating the dual-modality nanoprobes for 48 h. Figure 2c showed that no significant differences were observed between the dual-modality nanoprobe group and the Gd-DTPA group (*p* > 0.05). The results indicated that our dual-modality nanoprobes had no detrimental effect on macrophages survival. The *in vitro* risk assessments are consistent with data on the stability of the dual-modality nanoprobes (Supplementary 2.1). Consequently, the results of the toxic evaluation *in vitro* for the dual-modality nanoprobes were satisfactory.

### 2.3. Toxic Effects on the Immune System in Mice

#### 2.3.1. The Generation of Reactive Oxygen Species (ROS) as an Essential Regulator of Immunity

**Figure 3:**
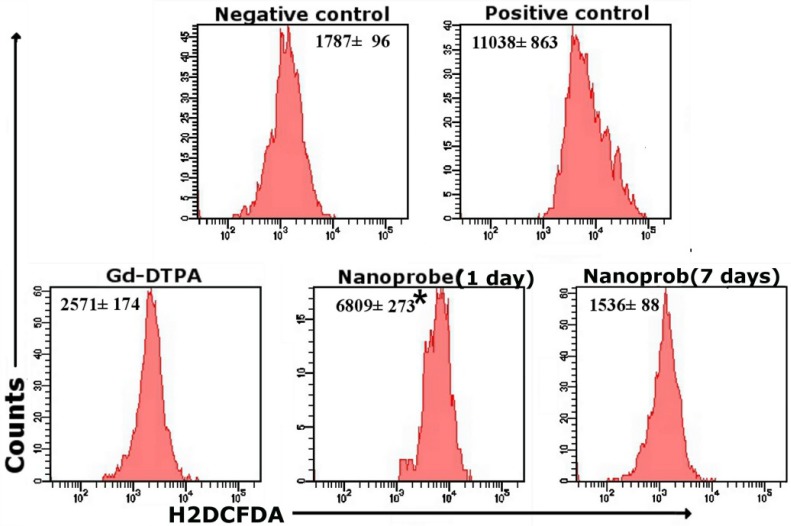
The generation of ROS in peripheral blood neutrophils was measured by flow cytometry 1 and 7 days after injection in Balb/c mice (20 μmol/kg, mean ± SD, *n* = 5). * *p* < 0.05 compared with the Gd-DTPA group.

Although the dual-modality nanoprobes showed promising biocompatibility *in vitro*, it is also important to evaluate their *in vivo* toxicity effects in mice because the culture conditions may not accurately recapitulate the *in vivo* immune system. Neutrophils in the peripheral blood are an important effector of host immunity. They provide first-line defense against infections [17]. Moreover, neutrophils in healthy individuals can produce up to 95% of circulating myeloperoxidase (MPO) that catalyzes the generation of ROS [18]. As a key regulator of immunity, ROS can trigger either immunosuppression or the eradication of pathogens during tissue-restoration [11]. Furthermore, we investigated the generation of ROS in neutrophils of the peripheral blood in Balb/c mice after injection. Figure 3 showed that the generation of ROS in peripheral blood neutrophils on the dual-modality nanoprobes group was 2-fold increased comparing to the Gd-DTPA group at 1 day. However, no significant differences were seen between the negative group and the dual-modality nanoprobe group after 7 days. The *in vivo* data showed that the dual-modality nanoprobes stimulated the generation of ROS at 1 day, and cells or tissues were affected by the oxidative stress to some degree. However, the level of ROS returned to the baseline at 7 days by physiological regulation. In many settings, ROS is relative to the host innate and adaptive immune response [19,20]. Therefore, we further studied the expression levels of certain CD markers and cytokines in the immune response at 1 day.

#### 2.3.2. The Expression of Representative CDs on the Immune Response

**Figure 4:**
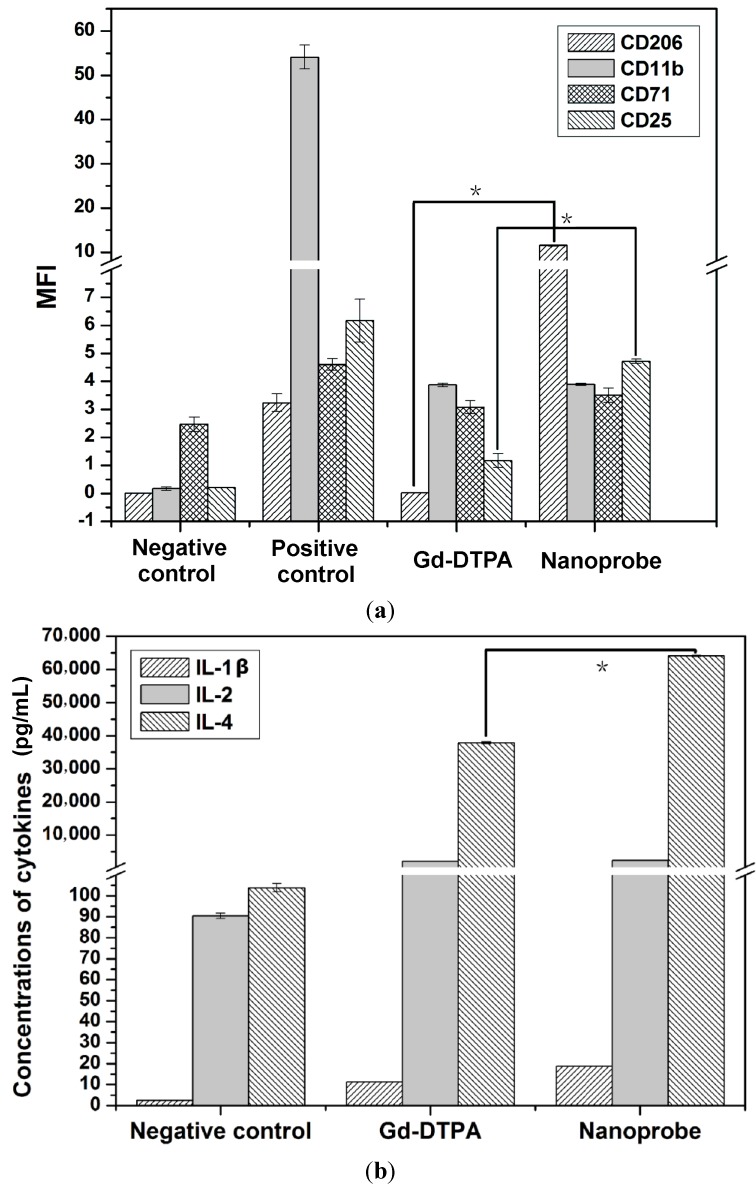
Toxicity of the immune response measured by flow cytometry 24 h after injection in Balb/c mice (20 μmol/kg, mean ± SD; *n* = 5). (**a**) The expression levels of CD206, CD11b, CD71 and CD25 in peripheral blood; and (**b**) The levels of IL-1β, IL-2 and IL-4 in serum. * *p* < 0.05 compared with the Gd-DTPA group.

CD206 is expressed on most tissue macrophages [21]; CD11b is mainly present on blood monocytes and immature macrophages [22]. CD25 and CD71 are surface markers in activated T cells [23]. Therefore, their expression levels are an important parameter in evaluating monocytes/macrophages and activated T cells. We investigated expression levels of CD markers from monocytes/macrophages in peripheral blood. The expression levels of CD206 and CD25 were significantly increased from the nanoprobe group compared to the Gd-DTPA group (Figure 4a and Figure S2). The number of macrophages and activated T cells was elevated. Importantly, expression levels of all the CD markers from the nanoprobe group are elevated *versus* the negative control group. The results indicated that our dual-modality nanoprobes might stimulate the immune cells via the over-generation of ROS after injection.

#### 2.3.3. The Secretion of Representative Cytokines on the Immune Response

To further elaborate on the *in vivo* immunotoxicity, we performed experiments on the secretion of representative cytokines. Because cytokines play an important role in modulating the host immune system, it is important to investigate how their expression might change in mice [24]. Interleukin-1β (IL-1β) is an endogenous pyrogen and is involved in the inflammatory response. The IL-1β stimulates thymocyte proliferation by inducing IL-2 release [25]. Interleukin-2 (IL-2) is a T-cell growth factor, and a required protein for T-cell proliferation and other regulators of the immune response [26]. Interleukin-4 (IL-4) has many biological roles including the stimulation of T-cell proliferation and B-cell activation [27].

The levels of these cytokines are presented in Figure 4b. We observed that there is a significant difference in the secretion of IL-4 between the nanoprobe group and the the Gd in a diethylenetriaminepentaacetic acid (DTPA) chelator (Gd-DTPA) group (*p* < 0.05). Moreover, the secretion of IL-2 and IL-4 from the nanoprobe group was increased *versus* the negative control group. The results indicated that some of the T-cells or B-cells were activated by the nanoprobes in mice, and T-cells might be stimulated to proliferation. But the immunotoxicity cannot be regulated endogenously. Consequently, it is important to evaluate immunotoxicity *in vivo* when studying the risk assessment of the rare earth-based nanomaterials.

### 2.4. Biodistribution of the Dual-Modality Nanoprobes at the Subcellular Level

**Figure 5:**
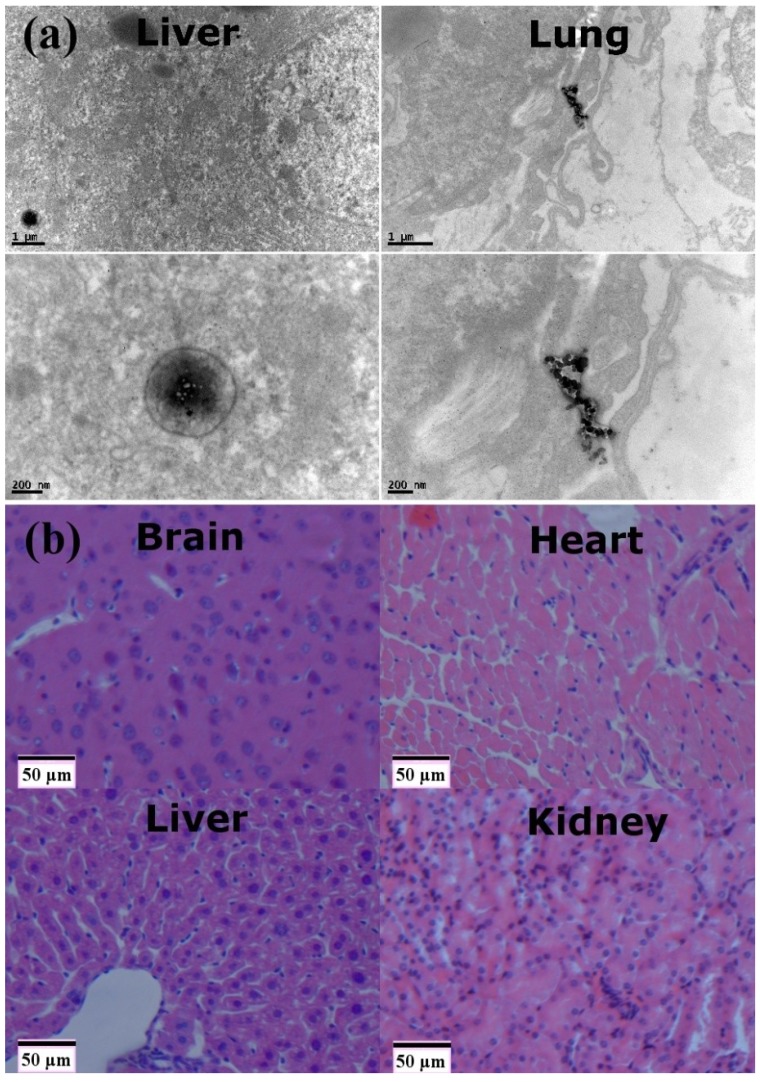
(**a**) The biodistribution of the dual-modality nanoprobes at the subcellular level. transmission electron microscopy (TEM) images from the liver and lung in mice 24 h after intravenous administration; and (**b**) Pathological biopsy assay on brain, heart, liver and kidney on the dual-modality nanoprobes stained with hematoxylin and eosin (H&E) and prussian blue.

The biodistribution of nano-based biomedicine relates to their biocompatibility [28]. To further visualize the biodistribution of nanoprobes, we used TEM. Sections of the liver and lung in mice were observed 24 h after injection (Figure 5a). Few nanoprobes were located in the cytoplasm of epithelial cells in the liver and lung. The results show that our nanoprobes were dispersed inside the phagosomes of the tissues and exhibited aggregation, which is consistent with the other researches [29,30]. As such, the dual-modality nanoprobes did slight damage to the microstructure or ultrastructure of the cells, and this may induce some of the toxic effects *in vivo*.

### 2.5. Pathological Analysis of the Dual-Modality Nanoprobes

The pathological biopsy assay (Leica DMIRBE, Wetzlar, Germany) was conducted on major organs including brain, heart, liver, kidney, spleen and lung 24 h after injection. Samples were stained with H&E and prussian blue. As shown in Figure 5b and Figure S3, there were no abnormal changes in any histological sections. This kind of the pathological examination provides data on the gross pathologic evaluation. The histologic and microscopic examinations demonstrated that the nanoprobes had not any influence on the cellular integrity and tissue morphology. Therefore, it is inevitable for *in vivo* immunotoxicity assessment to evaluate the potential health risks on the rare earth-based biomaterials.

## 3. Experimental Section

### 3.1. Synthesis and Characterization of the Dual-Modality Nanoprobes

Dual-modality nanoprobes, SiO_2_@(Y_0.5_Gd_0.45_Eu_0.05_)_2_O_3_ nanocomposites, were prepared according to our previous report [13]. The characterization mainly used TEM (A JEM-2010 HR, JEOL Ltd., Tokyo, Japan) and scanning electronic microscopy (SEM) (LEO-1530VP, LEO, Oberkochen, Germany). The T1-weighted MRI and the relaxivity (*r*_1_) were measured by a 1.5 T MRI scanner (General Electric, Sigma, Milwaukee, WI, USA).

### 3.2. The in Vitro Biocompatibility of the Dual-Modality Nanoprobes

#### 3.2.1. Cytotoxicity Assay

Murine macrophage RAW264.7 cells were cultured with Dulbecco’s Modified Eagle Media (DMEM, Gibco, Grand Island, NY, USA) and 10% fatal bovine serum (FBS, Gibco, Grand Island, NY, USA) in 96-well plates and incubated with different concentrations of nanoprobes (0.1 and 1 mM) during the logarithmic growth period for 24 and 48 h. Treatment with culture media and 0.5% dimethyl sulfoxide (DMSO, Sigma-Aldrich Corp., St. Louis, MO, USA) served as the negative control and the positive control, respectively. DMEM was removed carefully 44 h after incubation followed by 20 μL of 3-(4,5-dimethylthiazol-2-yl)-2,5-diphenyltetrazolium bromide (MTT, Sigma-Aldrich Corp., St. Louis, MO, USA) and incubated for 4 h followed by 100 μL dimethyl sulfoxide (DMSO) for 10 min with gentle shaking. Absorbance was measured at 490 nm using a microplate reader (Bio-Rad, San Diego, CA, USA).

#### 3.2.2. Apoptosis Assay

The murine macrophage RAW264.7 cells were incubated with DMEM and 10% FBS in 6-well plates and treated with PBS (phosphate-buffered saline; negative control), LPS (lipopolysaccharide; positive control, Sigma-Aldrich Corp., St. Louis, MO, USA), the Gd in a diethylenetriaminepentaacetic acid (DTPA) chelator (Gd-DTPA) (commercial MRI contrast agent; 1 mM, Magnevist, Berlex Laboratories, Wayne, NJ, USA) and dual-modality nanoprobes (0.1 and 1 mM) for 48 h. We then removed the culture media and digested the cells with 0.5% trypsin-0.2% EDTA (Ethylene diamine tetraacetic acid) (Sigma-Aldrich Corp., St. Louis, MO, USA) for 3 min. Cells were washed twice with cold PBS by gentle shaking. Samples were stained with Annexin-V/PI (propidium iodide) (kit from eBioscience, San Diego, CA, USA). We resuspended the cells in 200 μL binding buffer (1×) at 1 × 10^6^ cells/mL and then added 5 μL Annexin V-fluorescein isothiocyanate isomer (FITC) into 195 μL of the cell suspension. This was mixed and incubated for 10 min at room temperature. Cells were washed twice with 200 μL binding buffer (1×) and resuspended in 190 μL binding buffer (1×). We then added 10 μL propidium iodide (PI) (20 µg/mL) into the cells. Finally, the samples were measured with a FACScan (Becton Dickinson, Mountain View, CA, USA).

### 3.3. In Vivo Immunotoxicity of the Dual-Modality Nanoprobes

Twenty male Balb/c mice were divided into four groups at random and injected via tail vein: (a) PBS (100 μL; negative control); (b) LPS (5 mg/kg); (c) Gd-DTPA (20 μmol/kg); and (d) the dual-modality nanoprobes (20 μmol/kg). Samples were obtained at 1 day after injection and measured by flow cytometry (Becton Dickinson, Mountain View, CA, USA).

#### 3.3.1. ROS Assay on Peripheral Blood Neutrophils

Peripheral blood from the tail vein (20 μL) was added to a tube with heparin sodium (4 μL). Erythrocytes were lysed by red blood cell lysis buffer (1 mL, Beyotime, Haimen, China) in the dark for 2 min. After removing the supernatant, PBS (2 mL) was added and the sample was centrifuged at 500× *g* for 5 min at 4 °C. We next removed the supernatant. The carboxy derivative of fluorescein, carboxy-H2DCFDA (C400), (H2DCFDA, Invitrogen, California, CA, USA) was added to the samples (1 × 10^6^ cells) at a final concentration of 5 µM. Cells were mixed thoroughly in the dark for 20 min and PBS (400 μL) was added.

#### 3.3.2. Expression Levels of Representative CD Markers in Peripheral Blood

Twenty microliters of peripheral blood from the ophthalmic vein was collected in a tube with heparin sodium (4 μL). The erythrocytes were lysed by red blood cell lysis buffer (1 mL, Beyotime, Haimen, China) in the dark for 2 min. We then removed the supernatant. Next, 1 × 10^6^ cells were stained in the dark for 30 min with anti-mouse CD206-PE, anti-mouse CD11b-PE, anti-mouse F4/80 allophycocyanion (APC), anti-mouse CD25-FITC, anti-mouse CD71-FITC and anti-mouse CD3-PE (Tianjin Sungene Biotech Co., Tianjing, China). This was followed by the addition of 2 mL PBS, centrifugation at 1200× *g* for 5 min at room temperature, and resuspension in 400 μL PBS.

#### 3.3.3. Levels of Cytokines in Serum

Peripheral blood was obtained by the vena ophthalmica in 1.5 mL eppendorf tubes. Samples were kept at room temperature for 45 min and then centrifuged at 3000× *g* for 10 min at 4 °C. The supernatant was transferred into new tubes and IL-1β, IL-2 and IL-4 levels were measured with ELISA kits (RayBiotech, Inc., Guangzhou, China) according to the provided instructions.

#### 3.3.4. Statistical Analysis

Data are means ± standard deviation (SD). Statistical analysis was performed by a one-way ANOVA and *t*-tests using Origin 8.0 software (OriginLab Co., Northampton, MA, USA). Data was considered significant when *p* < 0.05.

## 4. Conclusions

The dual-modality nanoprobe is a contrast agent for magnetic resonance/optical imaging and has satisfactory biocompatibility *in vitro* (Figure 2 and Figure S4). Unfortunately, the *in vivo* immunotoxicity is poor because of its nano-structure. The mechanism of immunotoxicity might be that the microstructure of the cells is slightly damaged by the overproduction of reactive oxygen species (ROS), which might stimulate the immune response in mice after injection. Therefore, there is no reason to study the nanoprobes with magnetic resonance imaging (MRI) *in vivo*, especially in light of the time and cost associated with MRI. This also suggests no need for clinical translation. It is very important for rare earth-based nanomaterials to be evaluated for immunotoxicity *in vivo*. This work suggests tools for evaluation of immunotoxicity *in vivo.*

## Supplementary Files

Supplementary File 1
