# Supplementary material for: In Vivo Immunotoxicity of SiO2@(Y0.5Gd0.45Eu0.05)2O3 as Dual-Modality Nanoprobes
Source: Int J Mol Sci. 2014 Aug 7;15(8):13649–62. doi: 10.3390/ijms150813649 (PMC4159817; doi:10.3390/ijms150813649)

Supplementary Information

1. Experimental Details

1.1. Cell Culture and Optical Imaging

RAW 264.7 cells were cultured at 37 °C under 5% CO_2 in DMEM with 10% FBS, penicillin (100 units/mL), and streptomycin (100 mg/mL). The cells were incubated with the dual-modality nanoprobes (0.06 mg/mL) at 37 °C, and 5% CO_2 for 2 h. They were then removed from remaining particles by washing with phosphate buffer solution (PBS), and detected under a laser scanning confocal microscope (Carl Zeiss AG, Oberkochen, Germany) at an excitation wavelength of 488 nm.

1.2. The Stability of the Dual-Modality Nanoprobes in Phosphate Buffer Solution (PBS)

Eight mM the dual-modality nanoprobes were placed into two tubes with 10 mL PBS (pH 7.4), respectively. Then divided into 5 tubes at random, the samples were covered and placed at 37 °C for 48 and 72 h, centrifuged at 15,000 rpm for 30 min. The supernatants were removed into the eppendorf tubes, and the concentrations of Gd^{3+} ions were detected by the Thermo Electron X7, ICP-MS (Thermo Instrument System Inc., St. Paul, MN, USA).

2. Results

2.1. Data on the Stability of the Dual-Modality Nanoprobes in PBS

The concentrations of Gd^{3+} ions in the supernatants were (0.010 ± 0.0002) and (0.016 ± 0.0003) nM at 48 and 72 h, respectively. The data suggested that there was nearly no free toxic Gd^{3+} ions dissociated from the nanoprobes in PBS at 48 and 72 h. Therefore, the stability of the dual-modality nanoprobes is satisfactory.

Figure S1. Raw data of Zeta potential on the dual-modality nanoprobes.

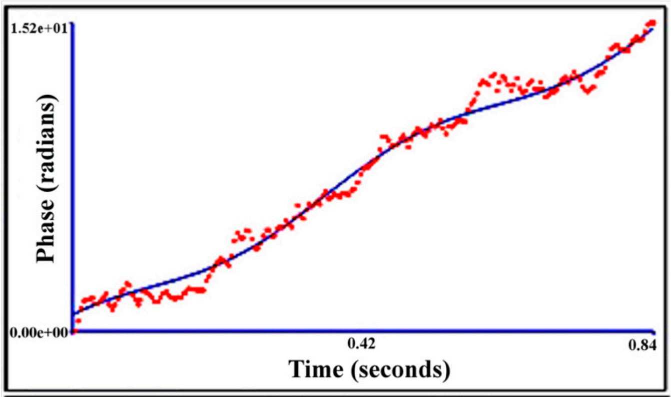

Run	Mobility	Zeta Potential (mv)	Rel Residual
1	-0.62	-7.93	0.0316
2	-0.19	-2.43	0.0291
3	-1.07	-13.70	0.0308
4	-0.27	-3.46	0.0375
5	-0.59	-7.58	0.0410
6	-0.68	-8.72	0.0602
7	-0.90	-11.56	0.0479
8	-0.25	-3.15	0.0245
Mean	-0.57	-7.31	0.0378
Std. Error	0.11	1.45	0.0041
Combined	-0.57	-7.29	0.0122

Figure S2. Histogram plot of CD206, CD11b, CD71 and CD25 in peripheral blood 24 h after injection in Balb/c mice (20 μmol/kg).

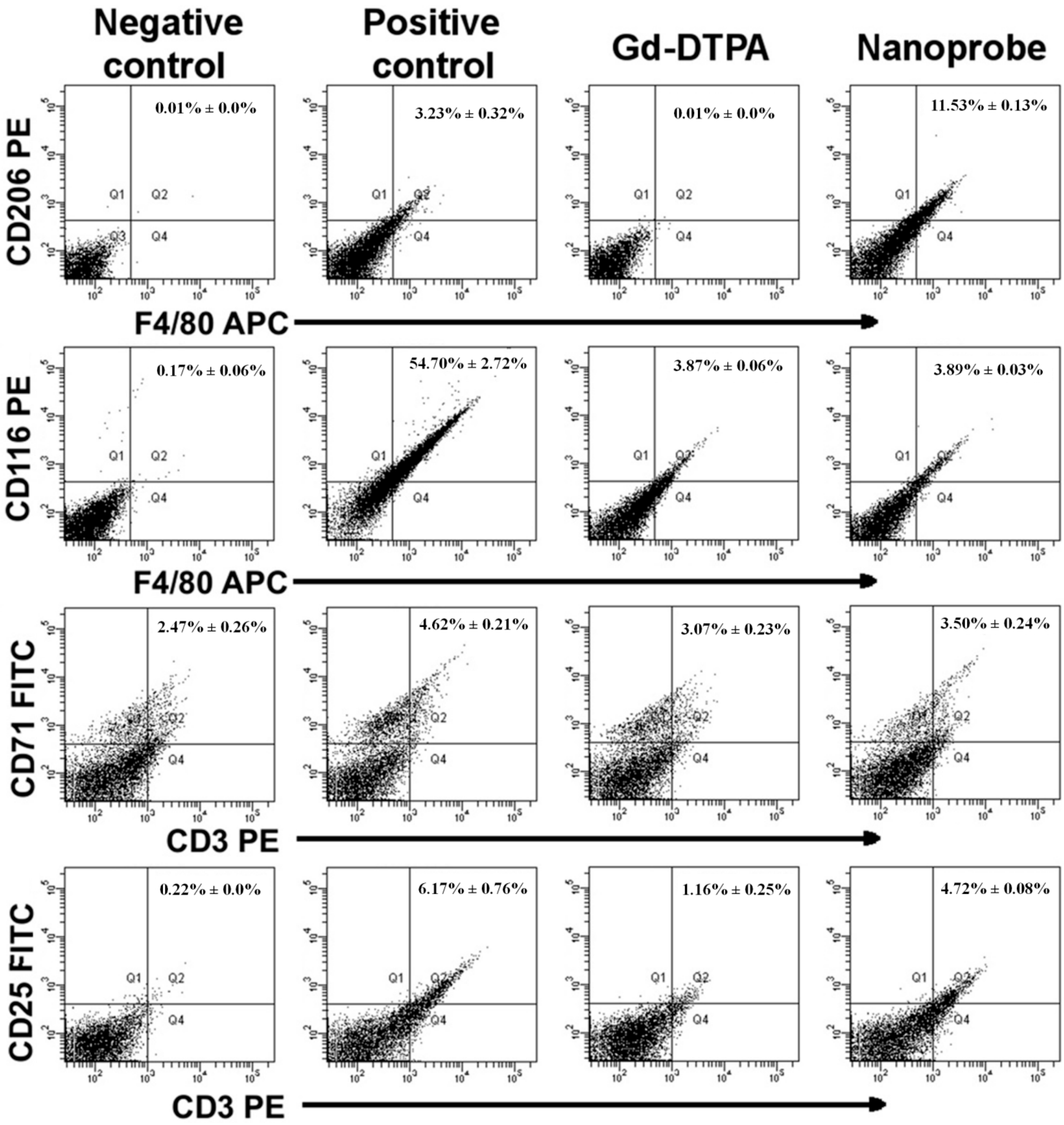

Figure S3. Pathological biopsy assay on spleen and lung on the dual-modality nanoprobes stained with H&E and prussian blue.

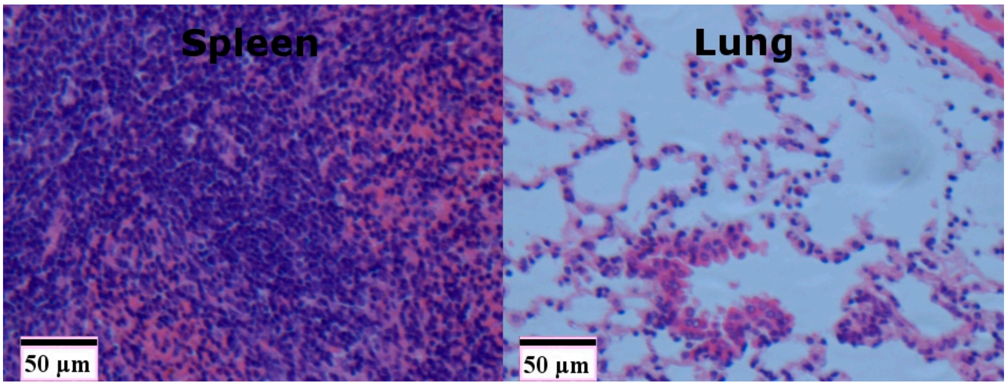

Figure S4. Confocal images of RAW 264.7 cells after 2 h of incubation with 0.06 mg/mL of nanoprobes. (**a**) Red fluorescence images; (**b**) blue fluorescence images; (**c**) bright-field images; and (**d**) superimposed images.

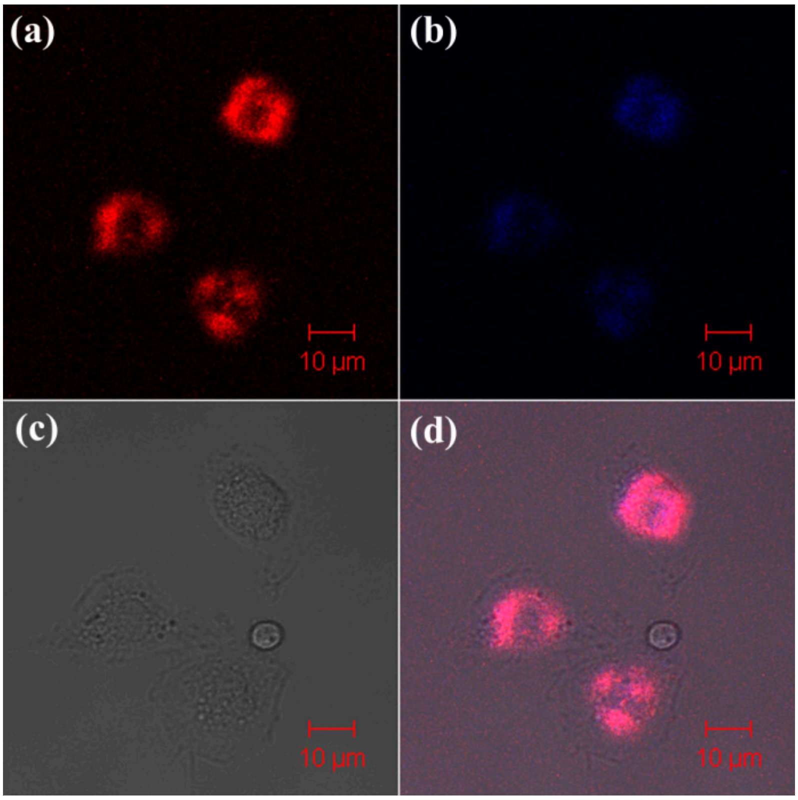

Supplement: Supplementary File 1 [file ijms-15-13649-s001.pdf]
